# Supplementary material for: Inhibition of Kv10.1 Channels Sensitizes Mitochondria of Cancer Cells to Antimetabolic Agents
Source: Cancers (Basel). 2020 Apr 9;12(4):920. doi: 10.3390/cancers12040920 (PMC7226288; doi:10.3390/cancers12040920)
Supplement: Supplementary file 1 [file cancers-12-00920-s001.zip › supplementary/cancers-766910-supplementary.docx]

**Supplementary Materials**

**Figure S1.** The absence of Kv10.1 induces mitochondrial fragmentation in HeLa KD and Du145 KD. (**a**–**d**) 3-D reconstruction from confocal stacks from HeLa (a,b) or DU145 (c,d) cells transfected with non-targeting siRNA (a,c) or siRNA against Kv10.1 (b,d) for 48 h, incubated with 100 nM Mitotracker Red (magenta, mitochondria) and 10 µg/mL Hoechst (cyan, nuclei) for 15 minutes and imaged using 0.2 µm z-step. The reconstruction was obtained using Imaris v9.0. The effect in Du145 cells is less evident due to the high degree of fragmentation in the control cells.

**Figure S2.** Tumor and Kv10.1 specificity of the effect of metabolic inhibitors. (a) The growth of non-tumor cells (hTERT-RPE1) was insensitive to metformin, phenformin or VLX-600 at the concentrations able to inhibit the growth of tumor cells (5 mM, 100 µM and 100 nM, respectively). (b) Lack of correlationof cytotoxicity with the expression level of Kv10.2 oor Kv11.1 in the different vell lines. The normalized cytotoxicity was plotted against the treshold values for the two channels in RT-PCR, analogously to Figure 8c.

**Figure S3.** Time course of Kv10.1 knockdown by siRNA. Cells transfected with non-targeting or Kv10.1-specific siRNA were harvested at the indicated times and the abundance of Kv10.1 (with respect to control siRNA) was determined by westernblot.
