# Supplementary material for: Inhibition of Kv10.1 Channels Sensitizes Mitochondria of Cancer Cells to Antimetabolic Agents
Source: Cancers (Basel). 2020 Apr 9;12(4):920. doi: 10.3390/cancers12040920 (PMC7226288; doi:10.3390/cancers12040920)
Supplement: Supplementary file 1 [file cancers-12-00920-s001.zip › supplementary/HernandezResendizSuppl.pptx]

## Slide 1
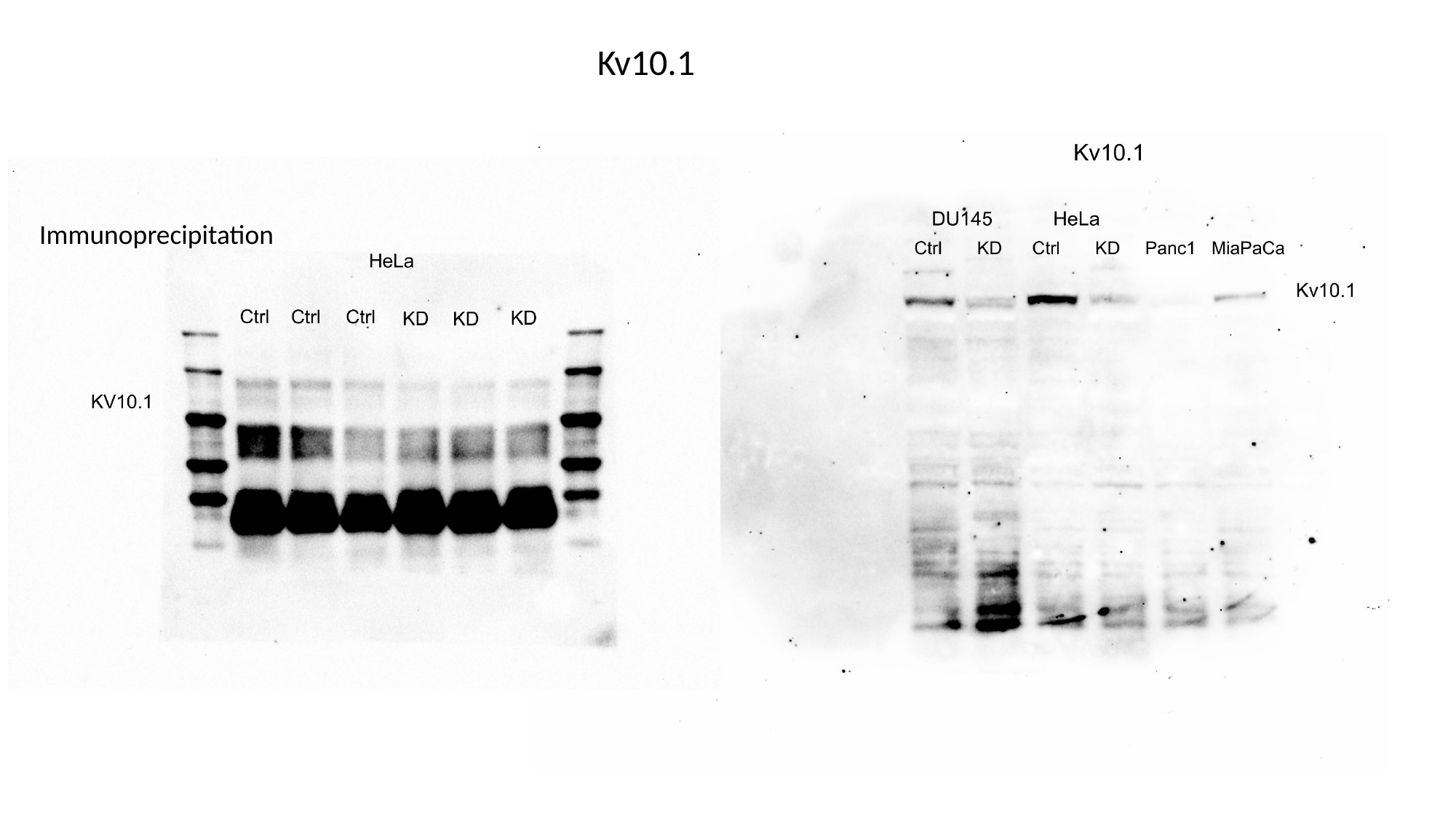

Kv10.1
Immunoprecipitation

## Slide 2
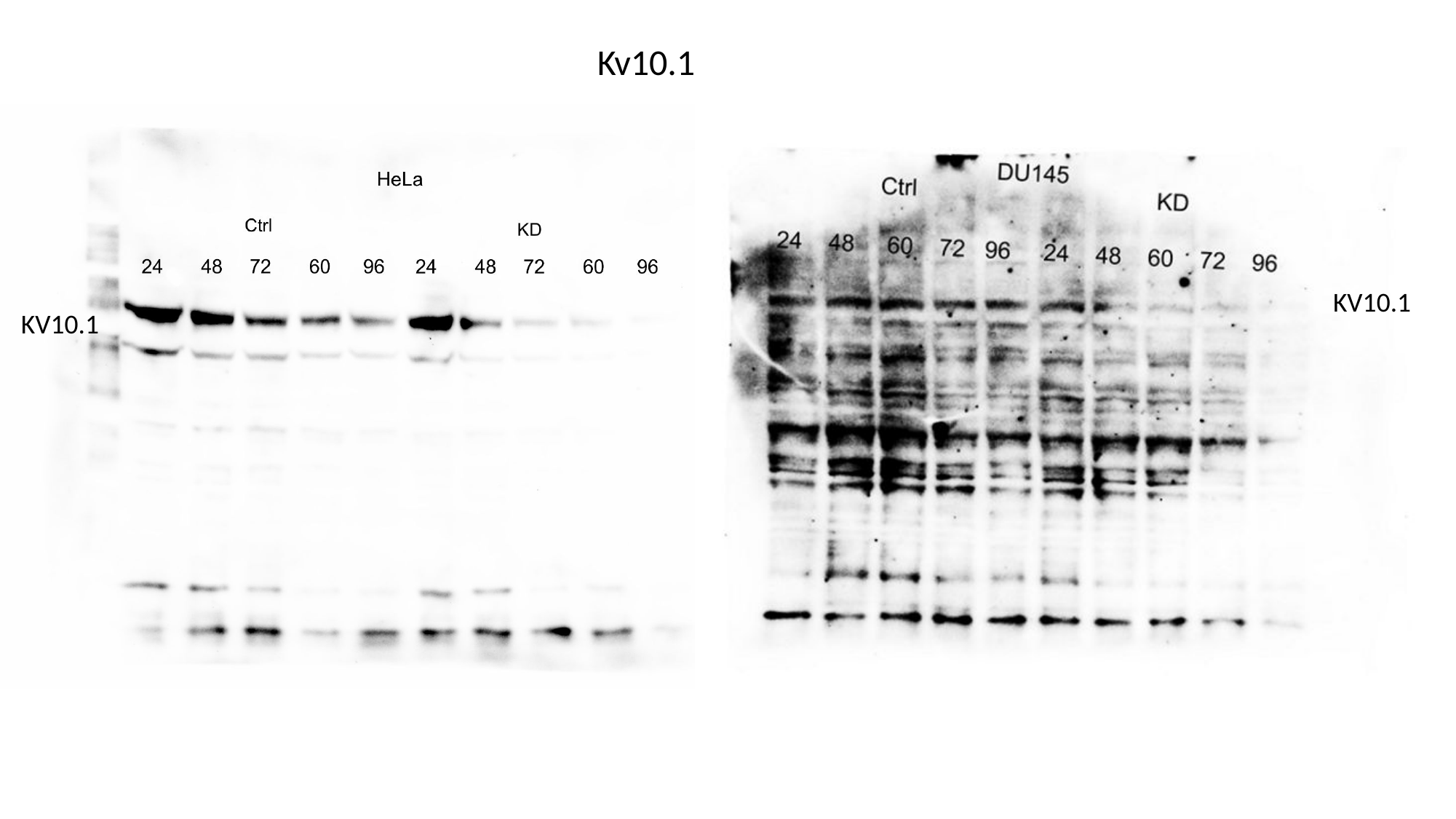

Kv10.1
KV10.1
KV10.1

## Slide 3
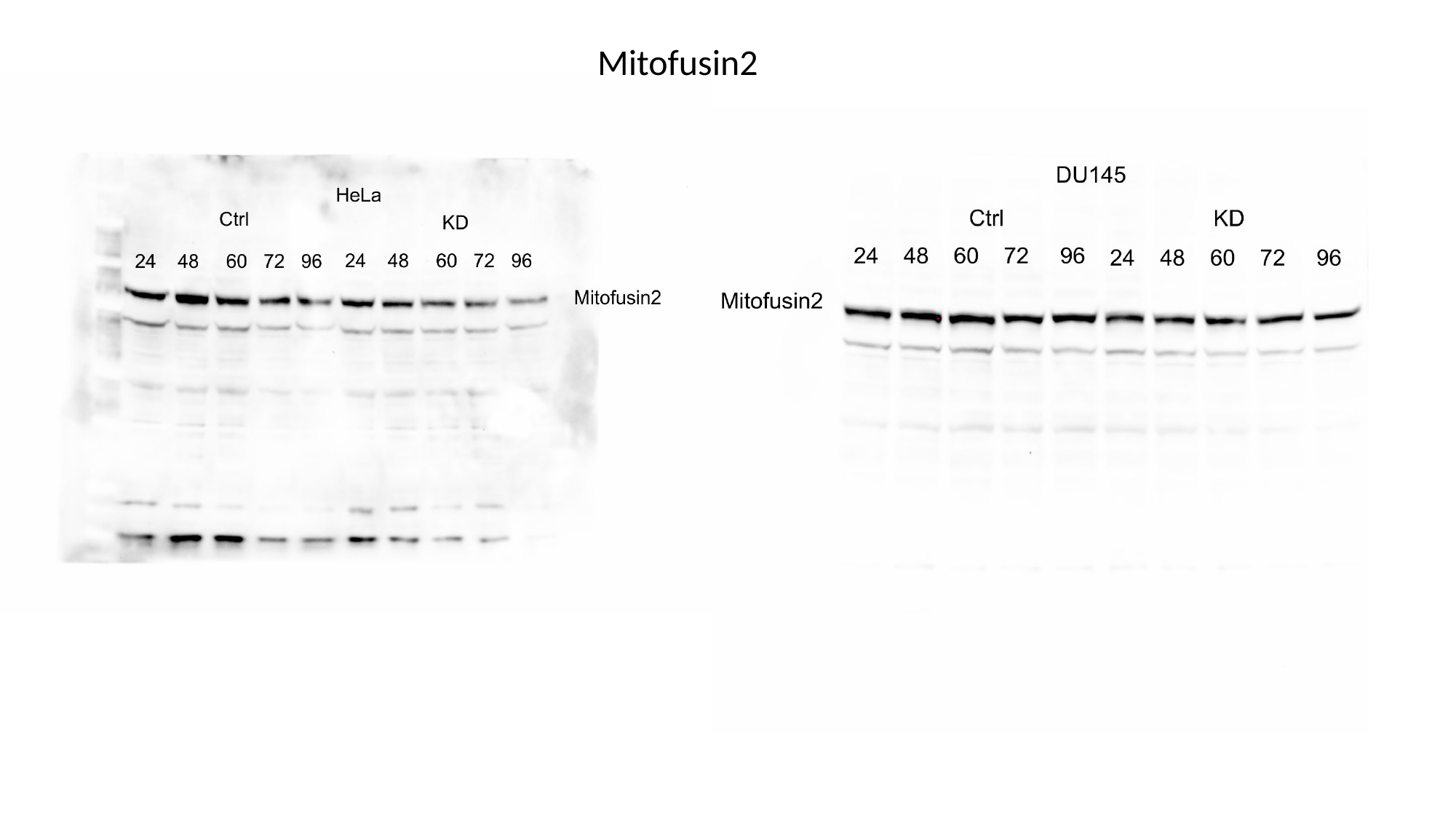

Mitofusin2

## Slide 4
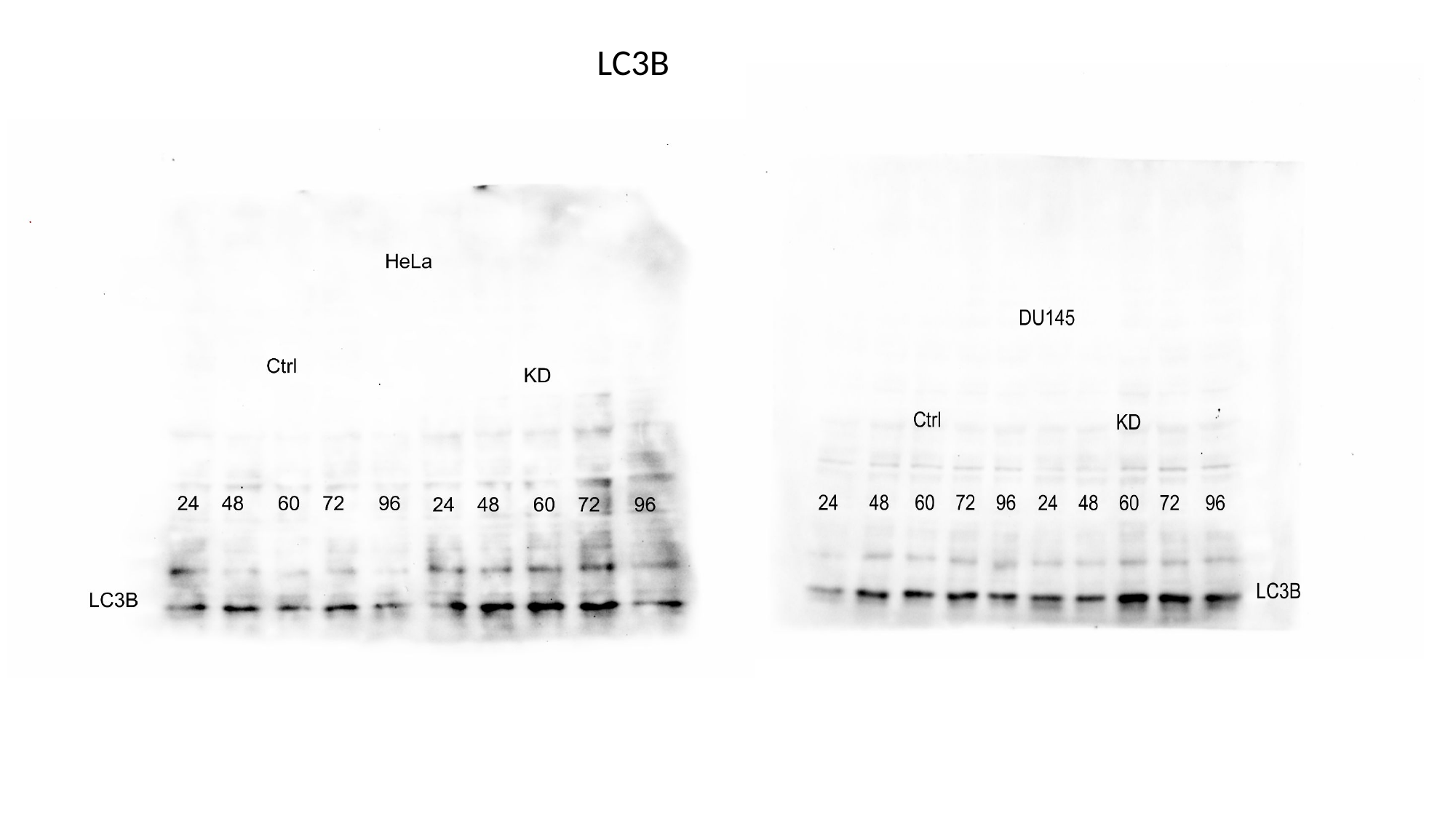

LC3B

## Slide 5
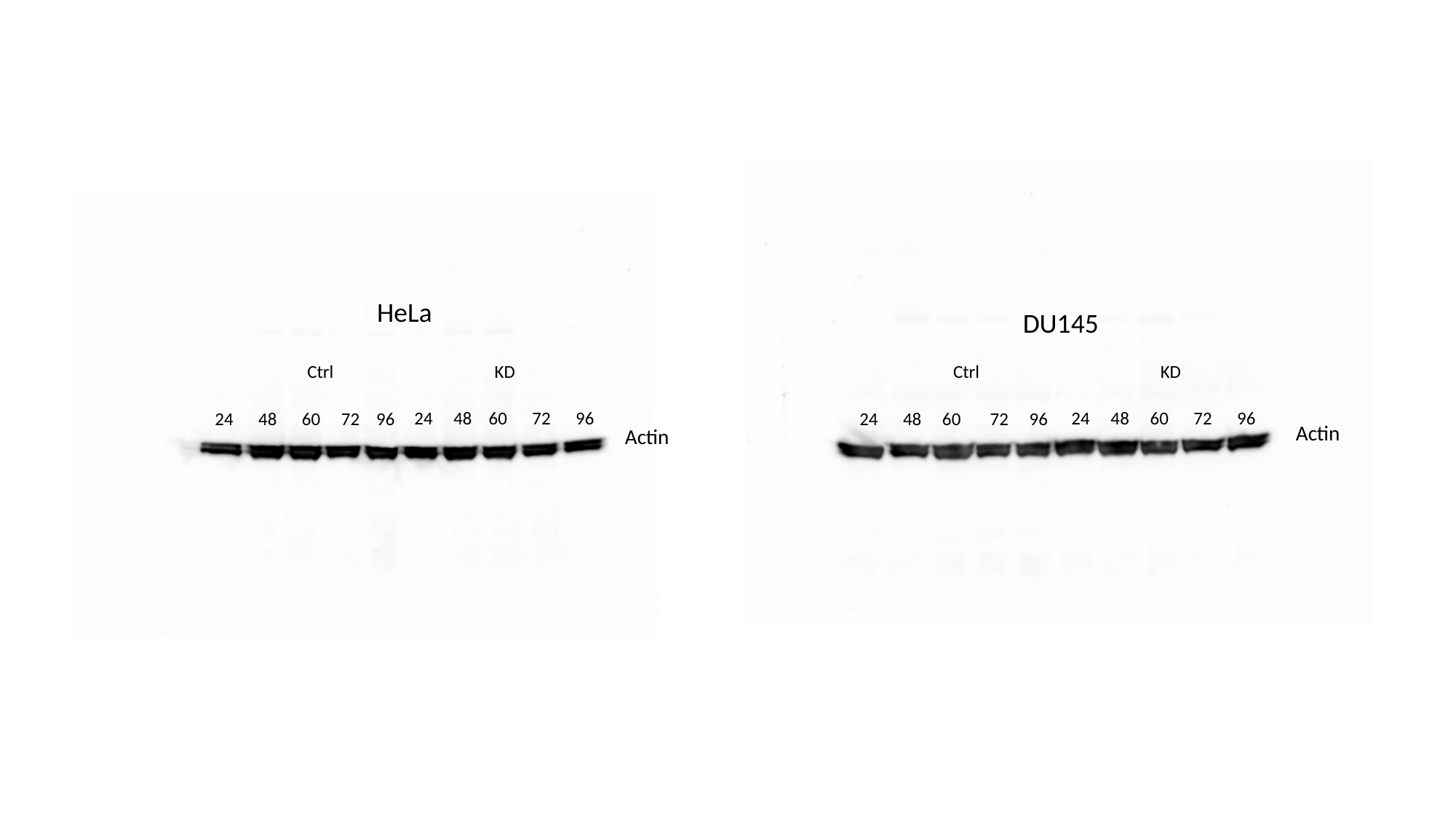

HeLa
DU145
KD
KD
Ctrl
Ctrl
24 48 60 72 96
24 48 60 72 96
24 48 60 72 96
24 48 60 72 96
Actin
Actin

## Slide 6
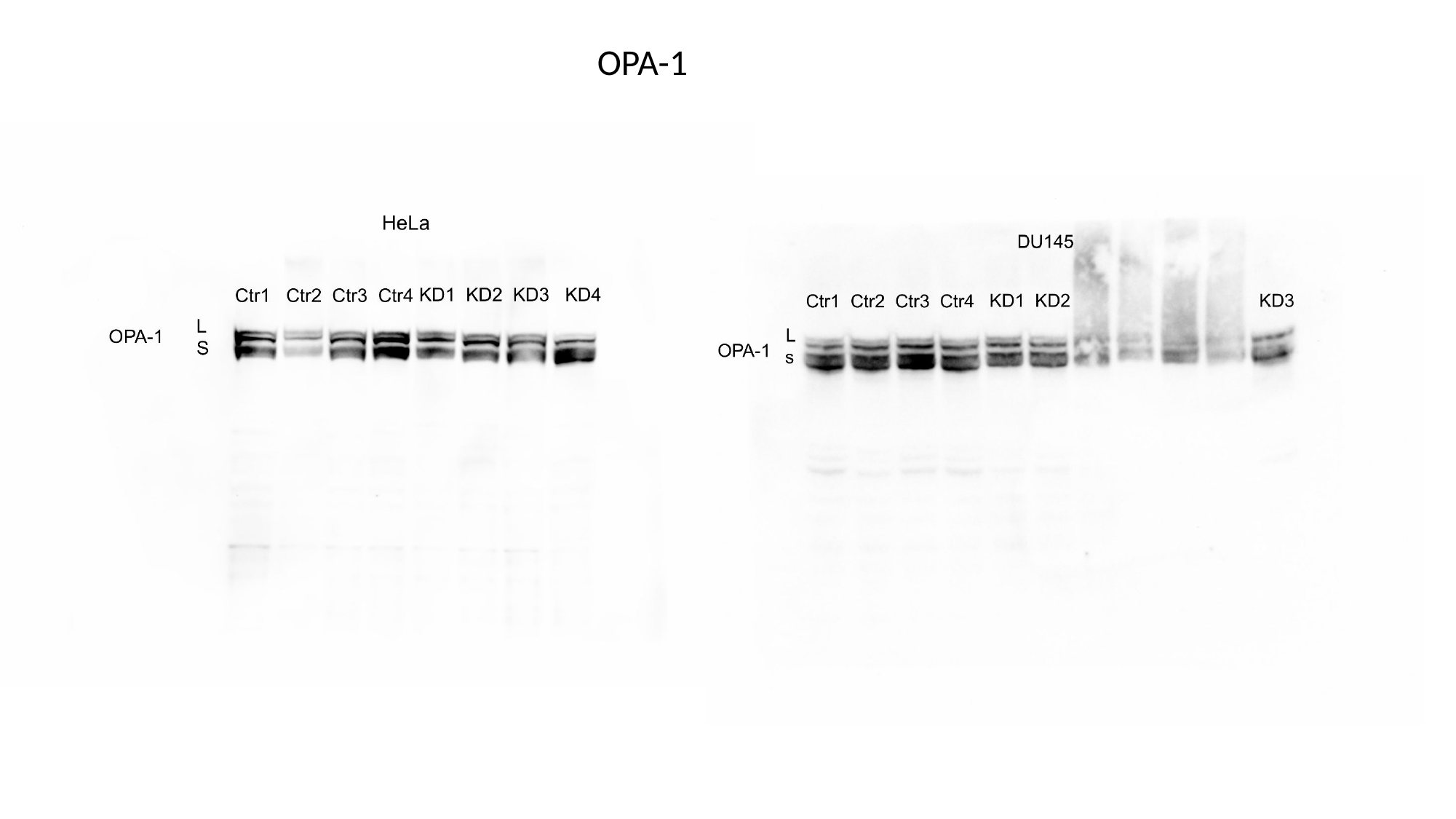

OPA-1

## Slide 7
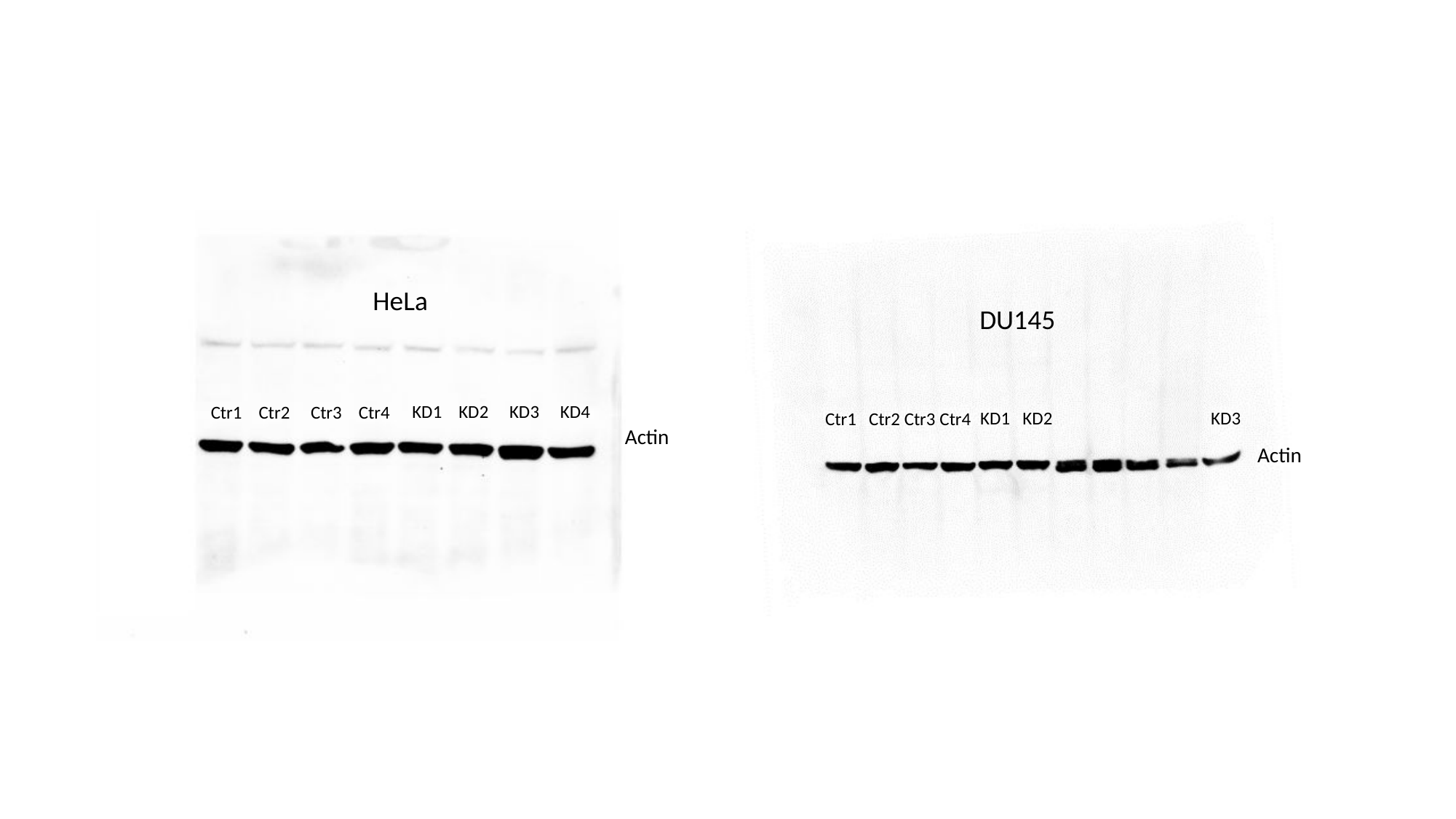

HeLa
DU145
KD1 KD2 KD3 KD4
Ctr1 Ctr2 Ctr3 Ctr4
KD1 KD2 KD3
Ctr1 Ctr2 Ctr3 Ctr4
Actin
Actin

## Slide 8
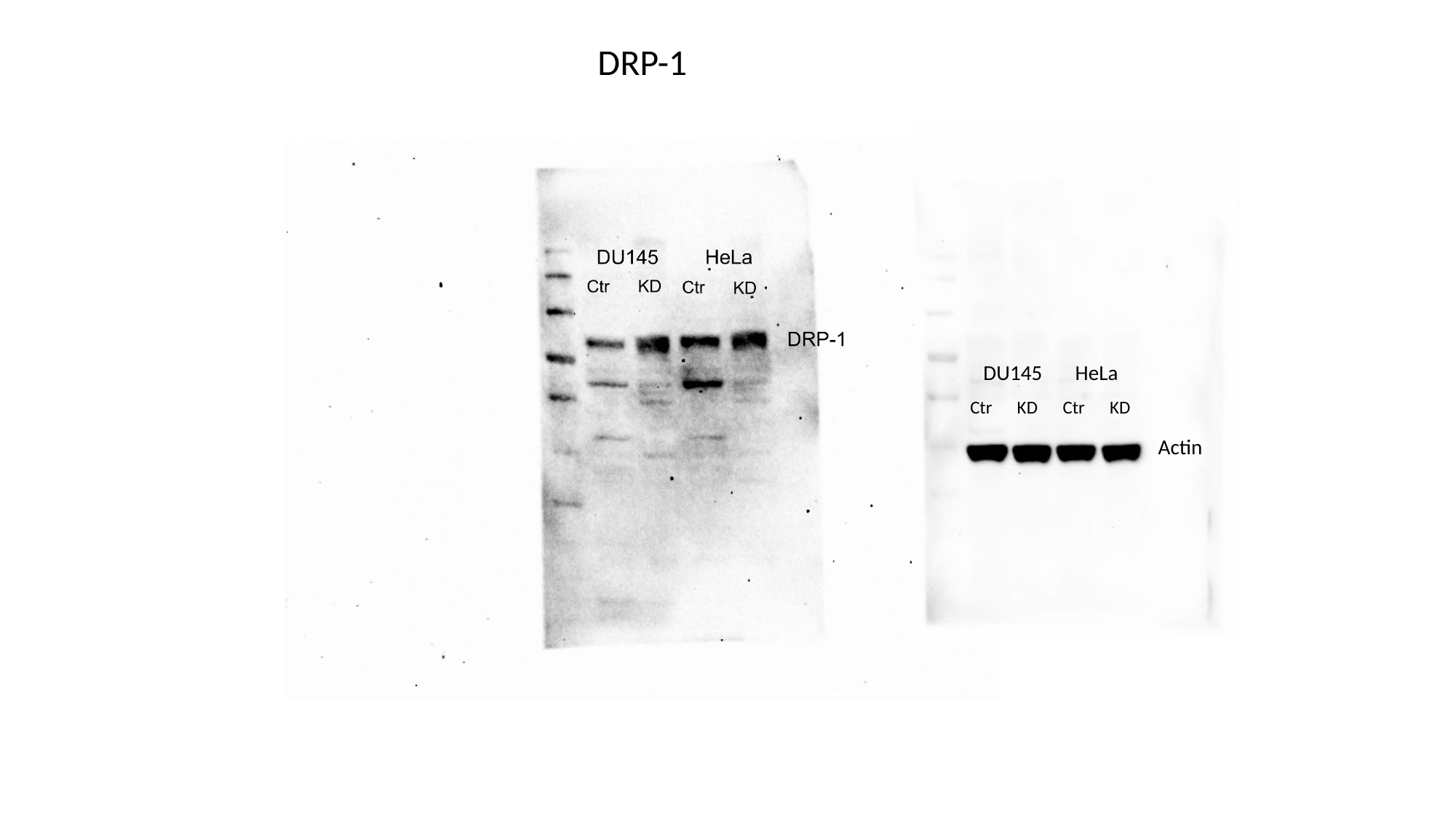

DRP-1
DU145 HeLa
Ctr KD Ctr KD
Actin

## Slide 9
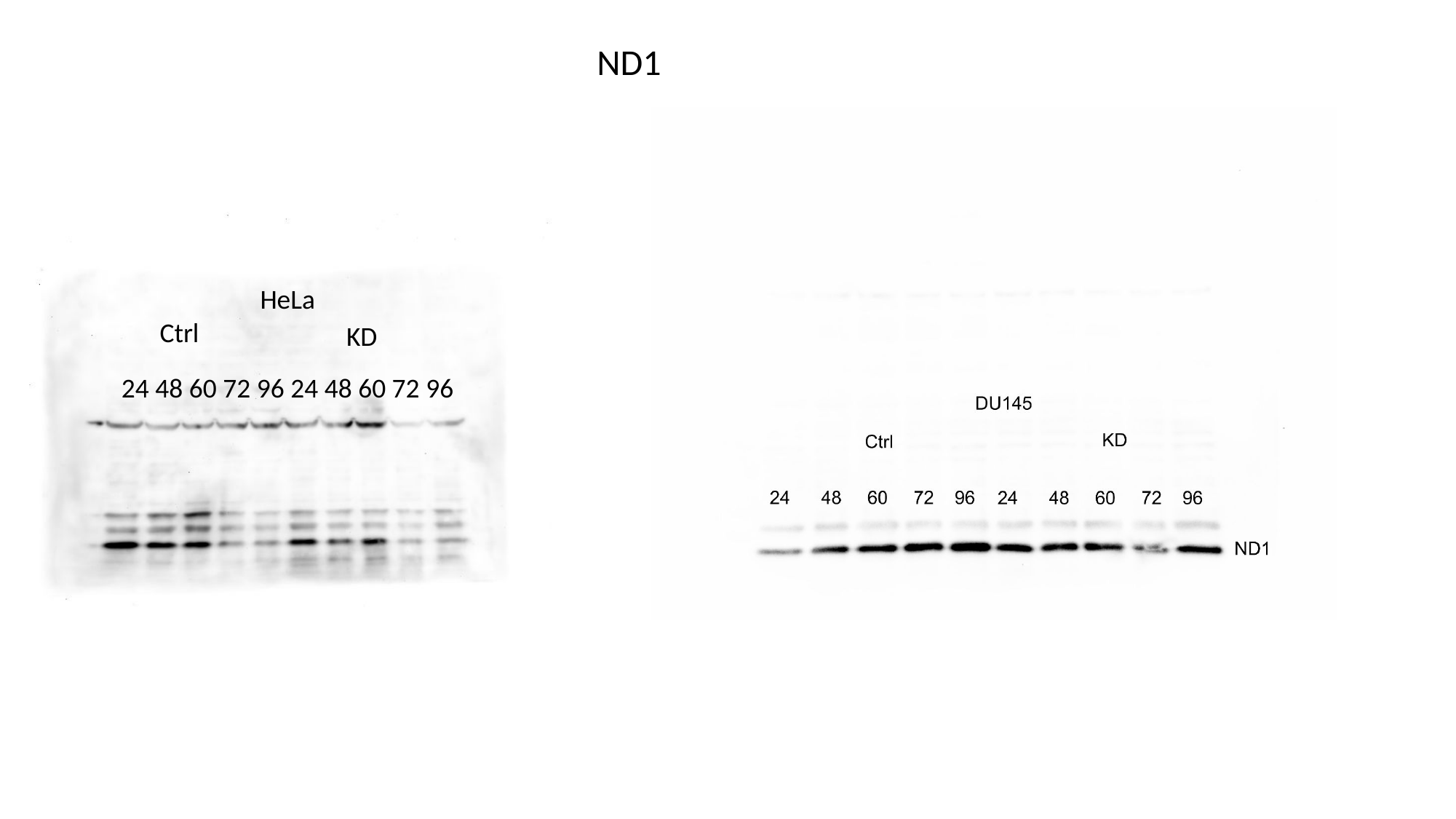

ND1
HeLa
Ctrl
KD
24 48 60 72 96 24 48 60 72 96

## Slide 10
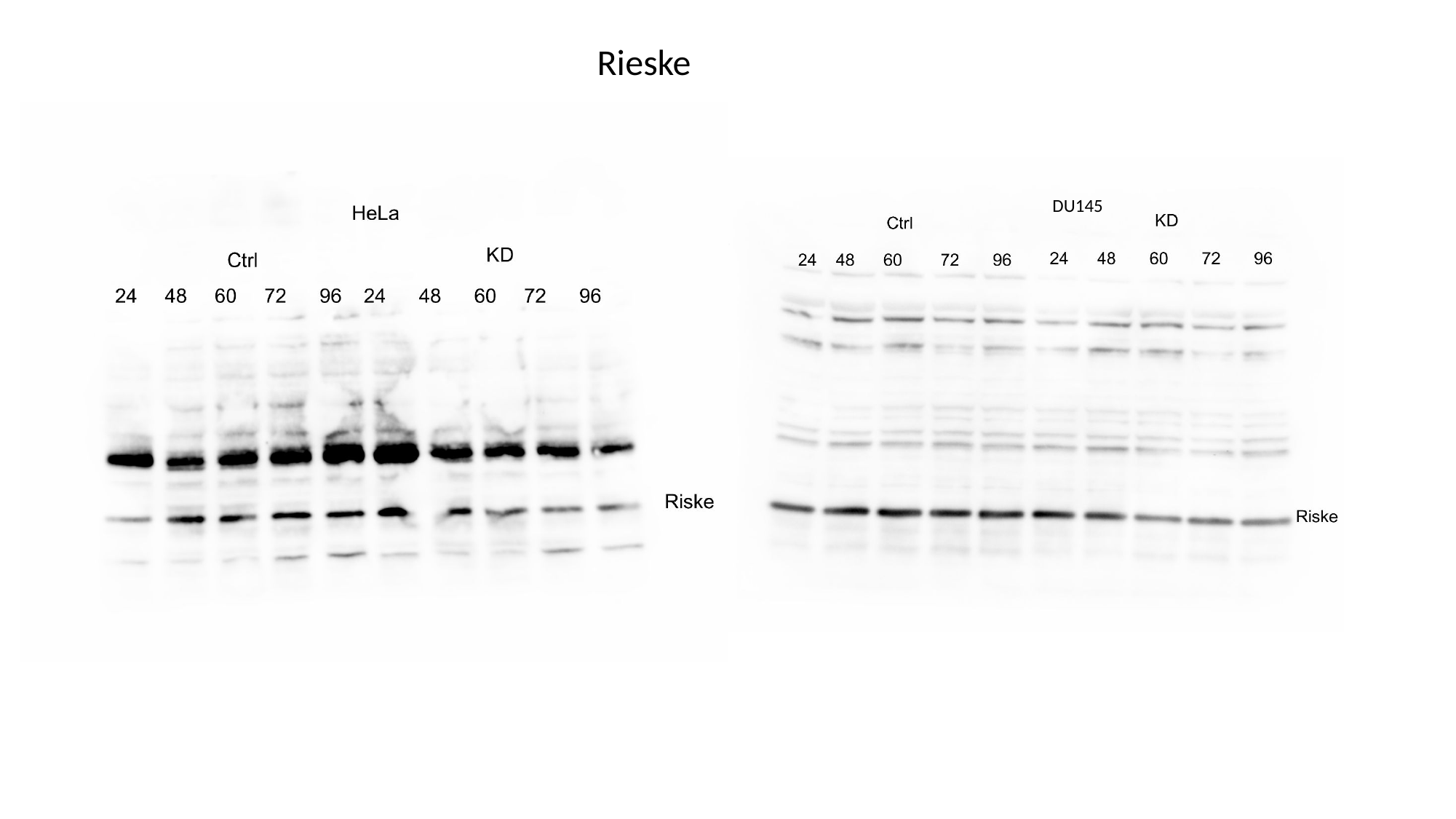

Rieske
DU145

## Slide 11
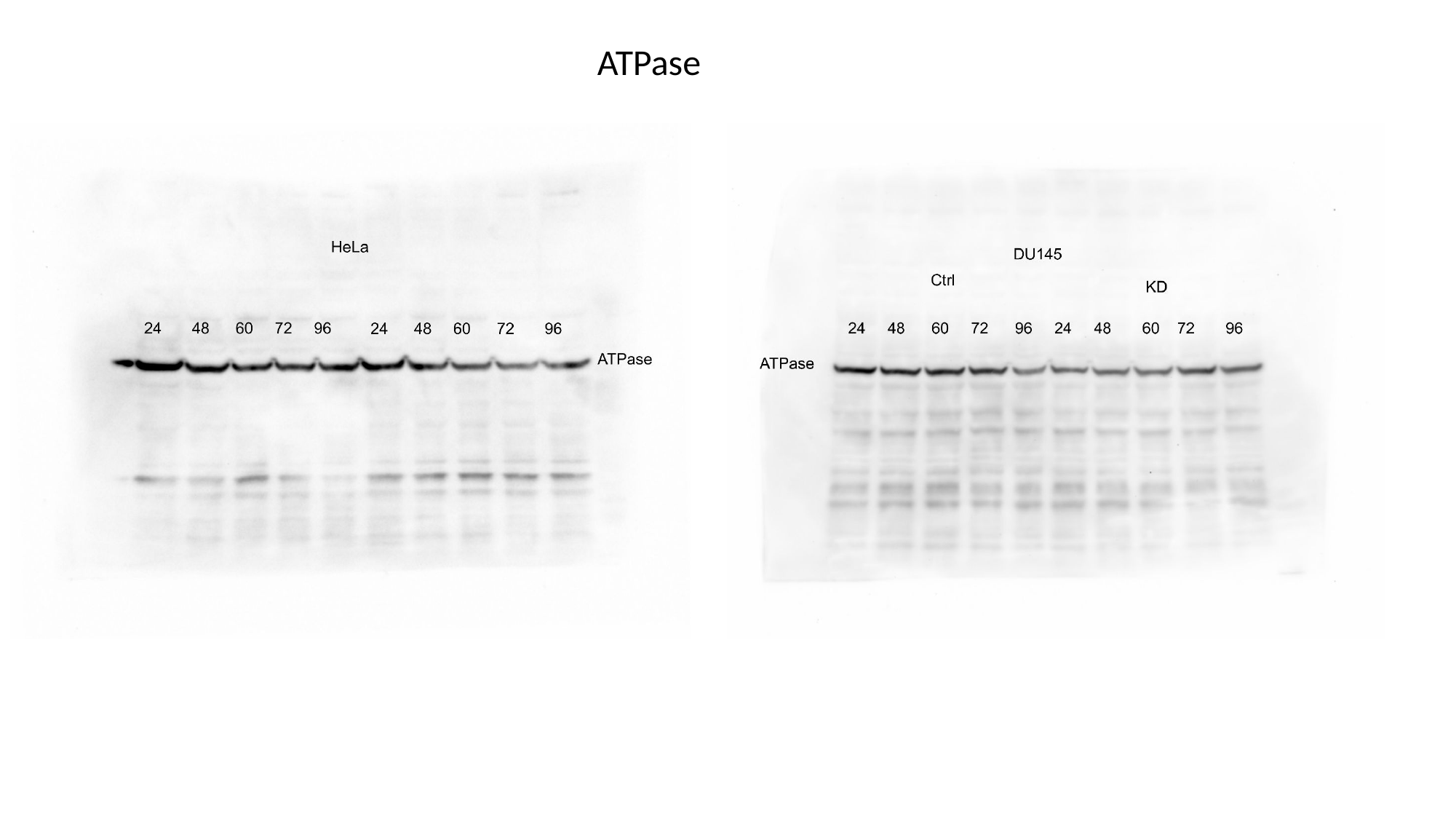

ATPase

## Slide 12
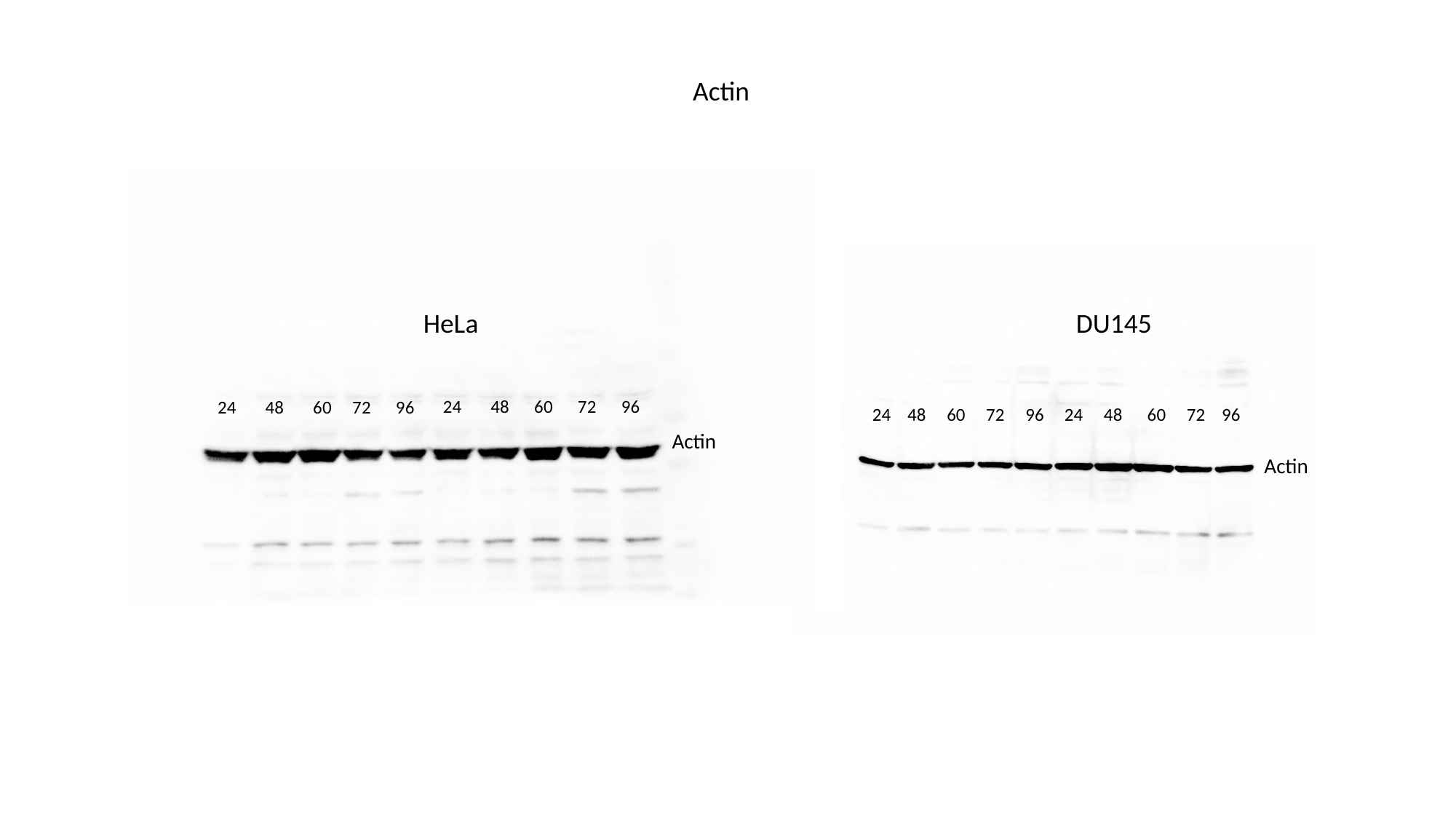

Actin
HeLa
DU145
24 48 60 72 96
24 48 60 72 96
24 48 60 72 96
24 48 60 72 96
Actin
Actin

## Slide 13
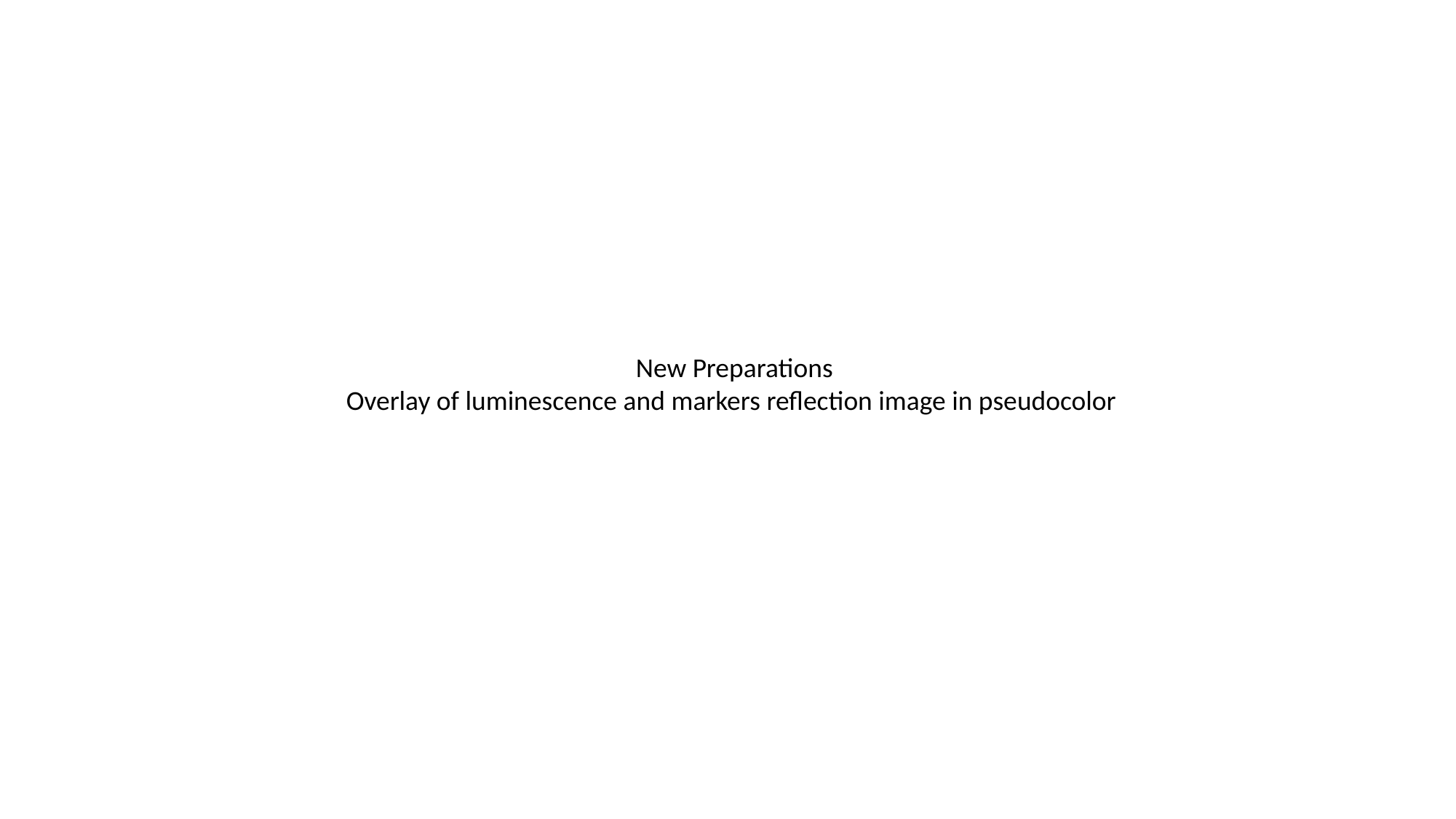

New Preparations
Overlay of luminescence and markers reflection image in pseudocolor

## Slide 14
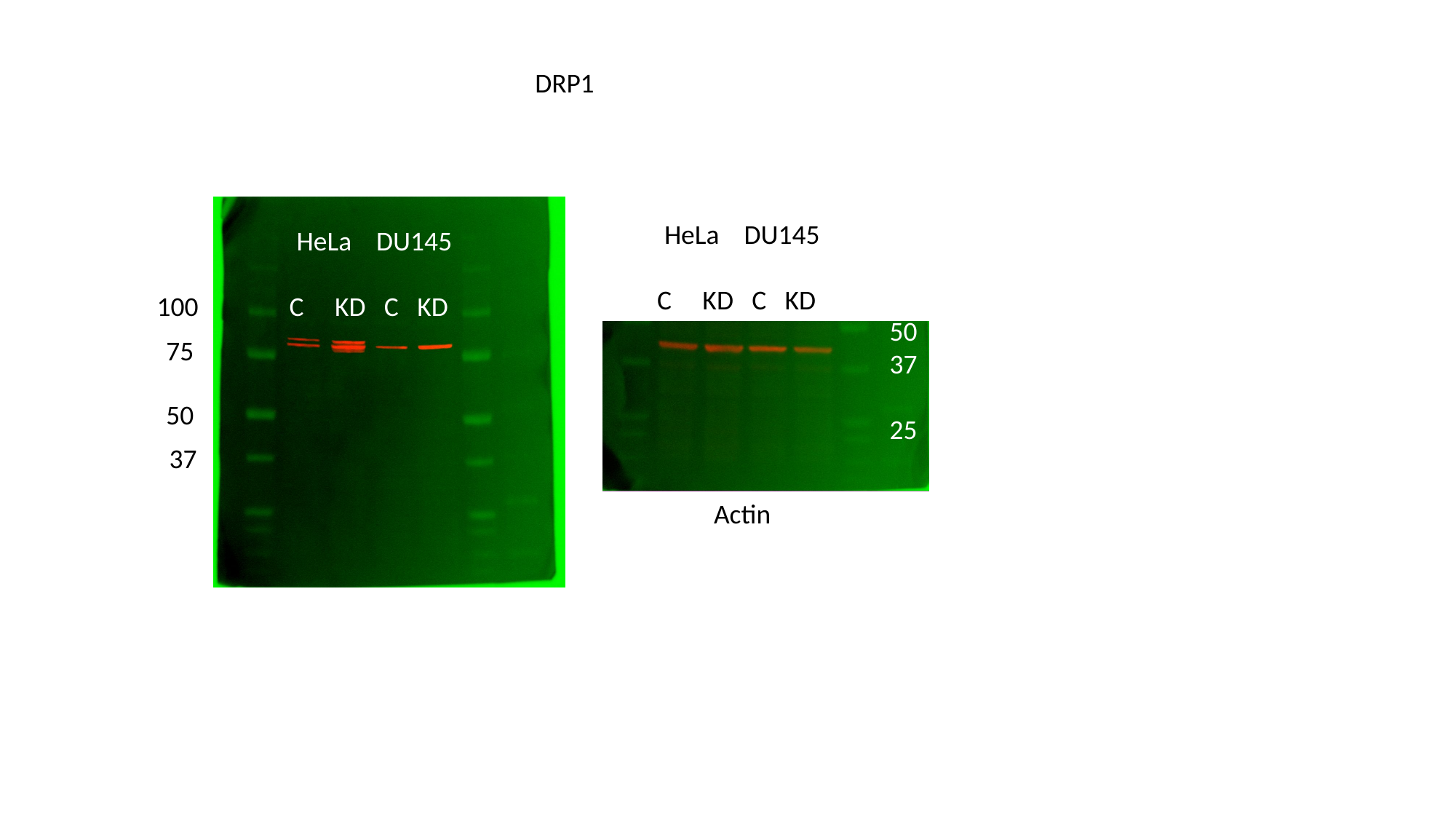

DRP1
HeLa DU145
HeLa DU145
C KD C KD
100
C KD C KD
50
37
25
75
50
37
Actin

## Slide 15
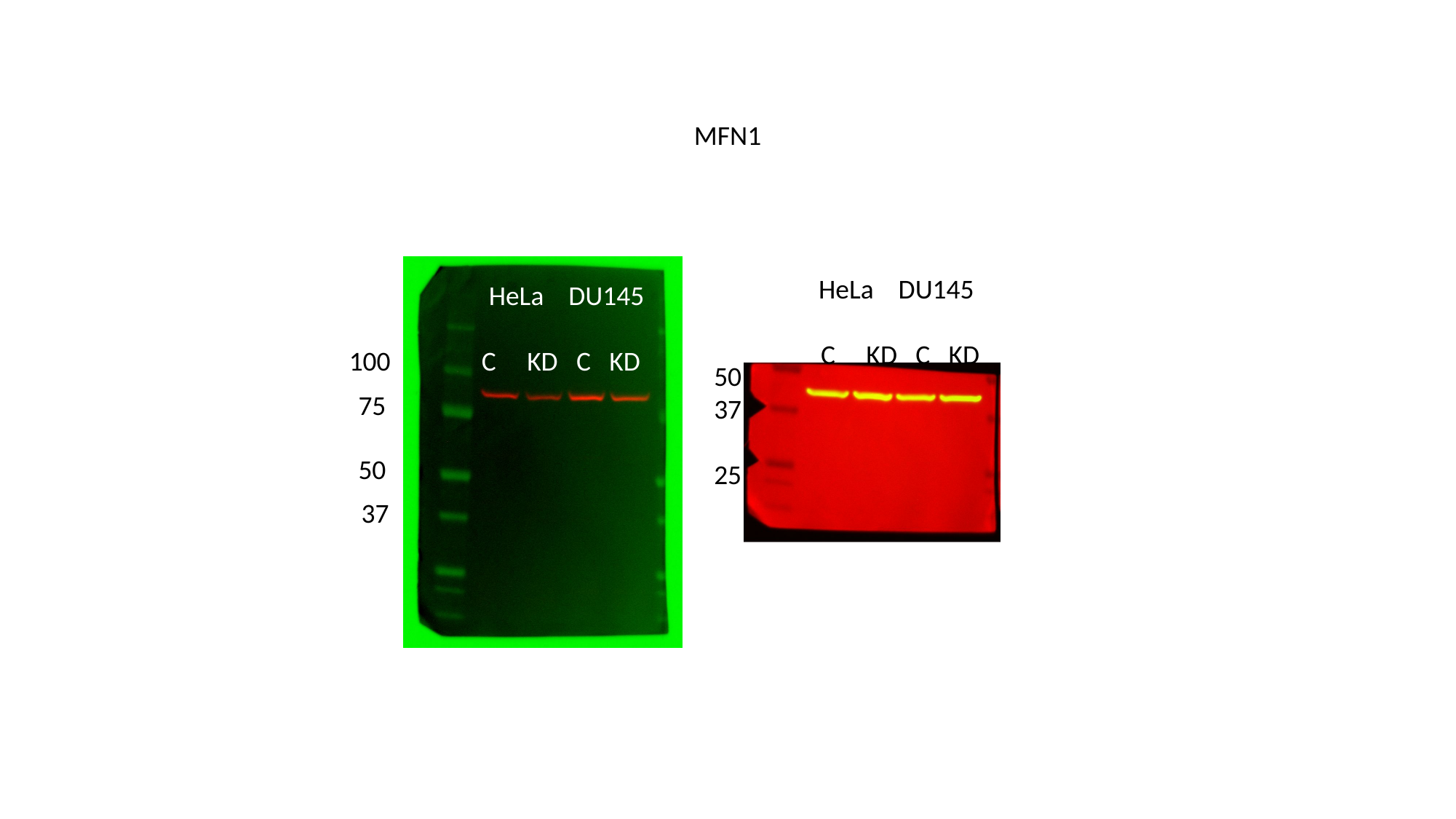

MFN1
HeLa DU145
HeLa DU145
C KD C KD
100
C KD C KD
50
37
25
75
50
37

## Slide 16
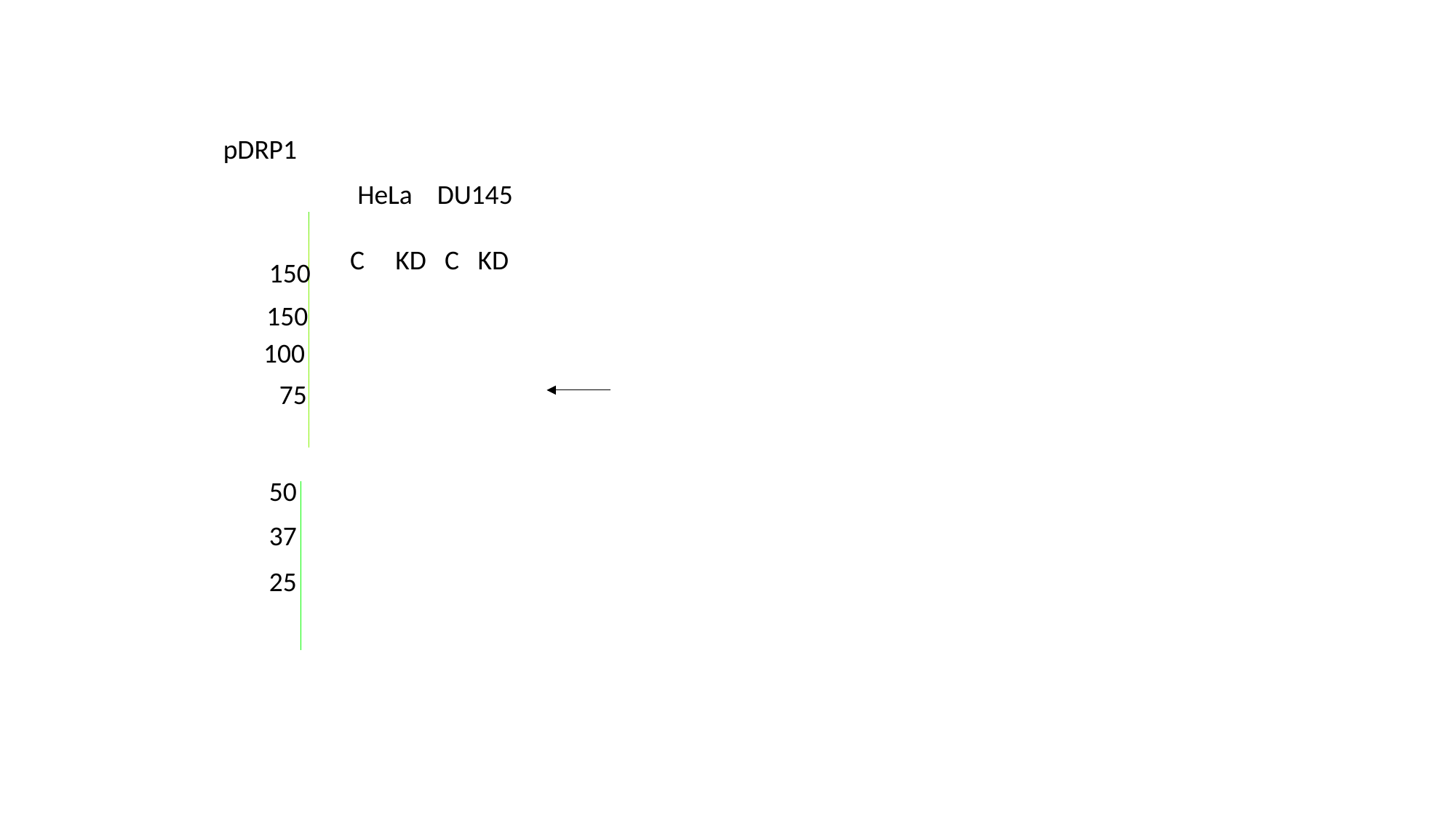

pDRP1
HeLa DU145
C KD C KD
150
150
100
75
50
37
25

## Slide 17
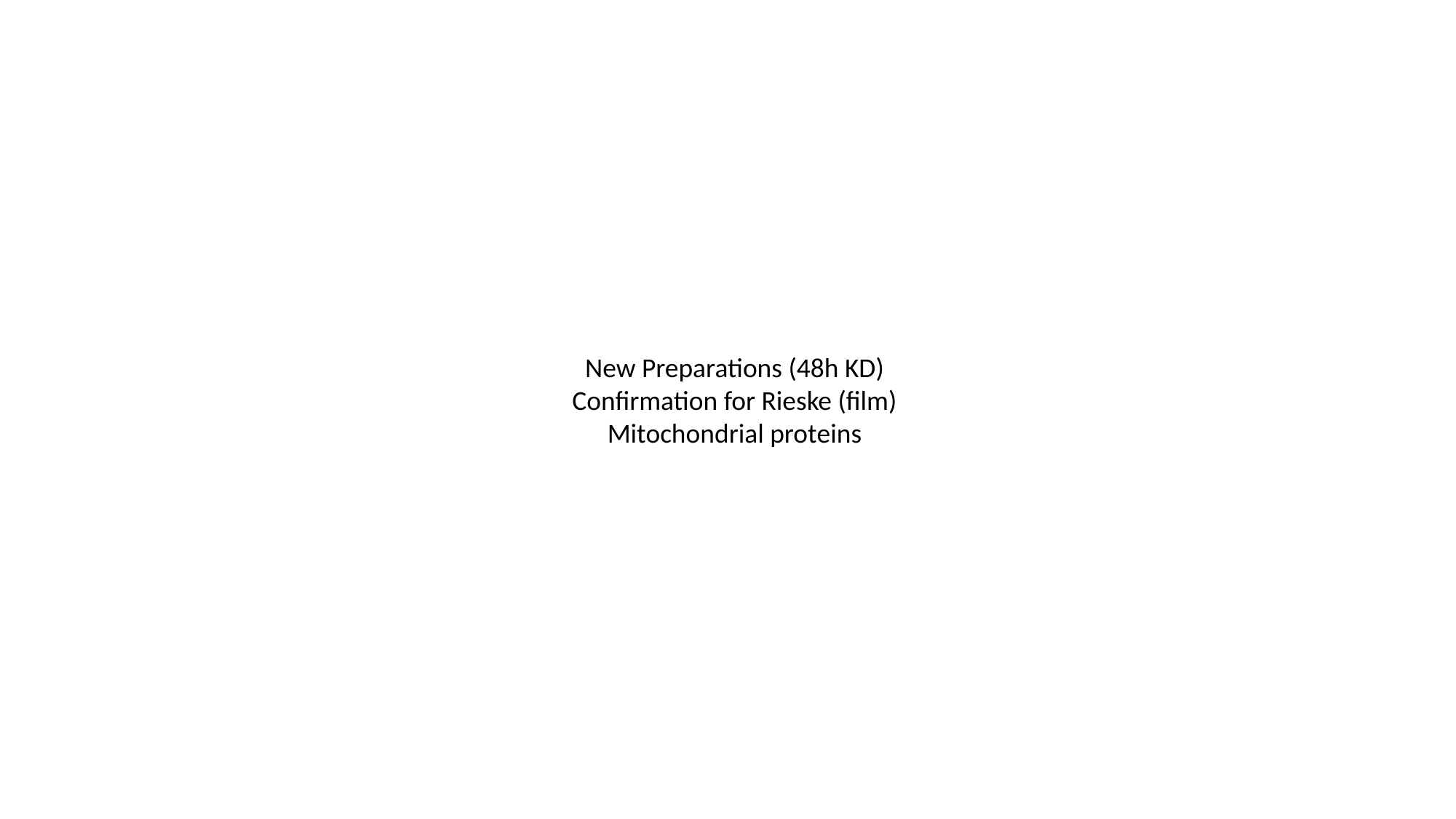

New Preparations (48h KD)
Confirmation for Rieske (film)
Mitochondrial proteins

## Slide 18
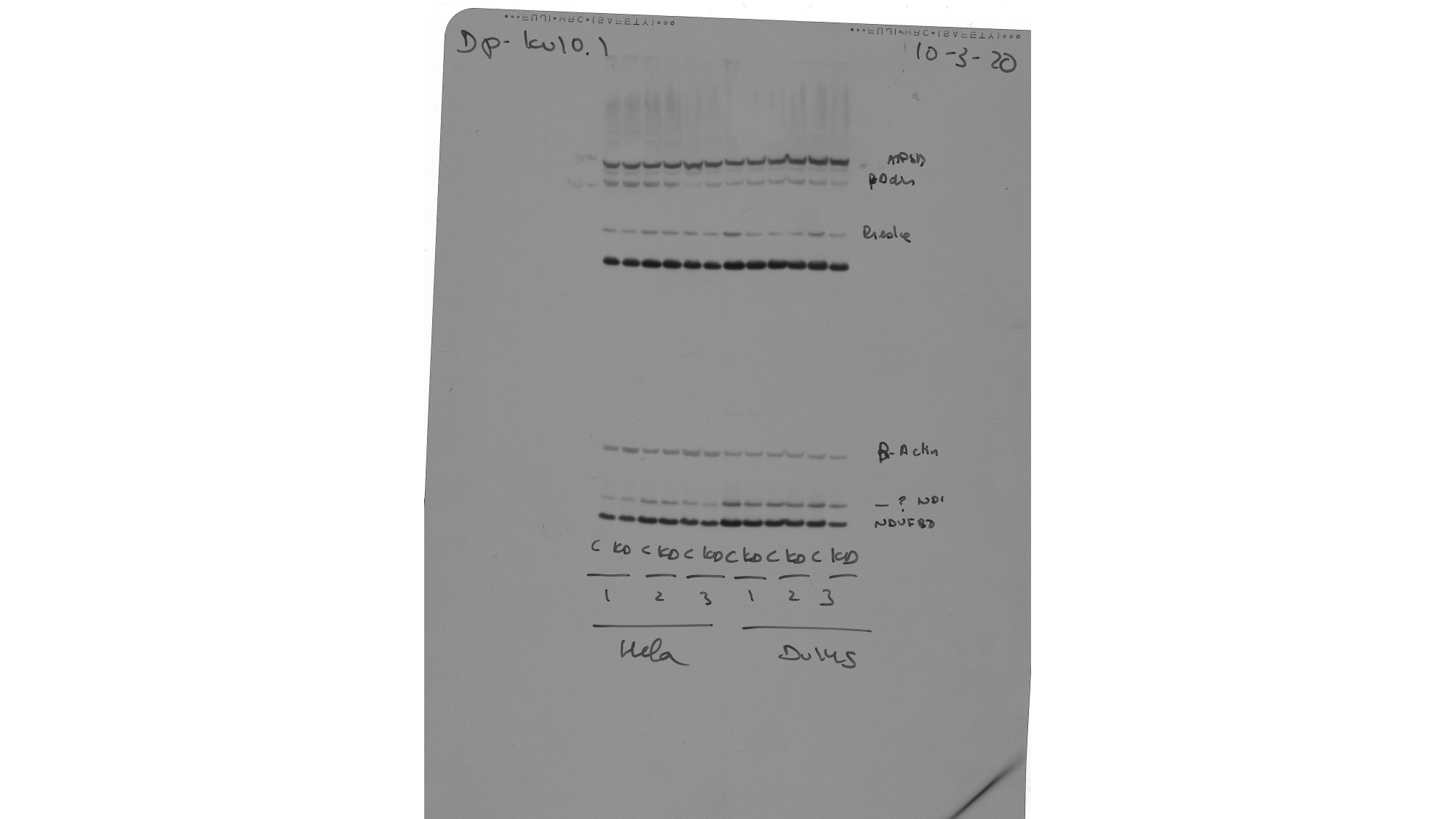

## Slide 19
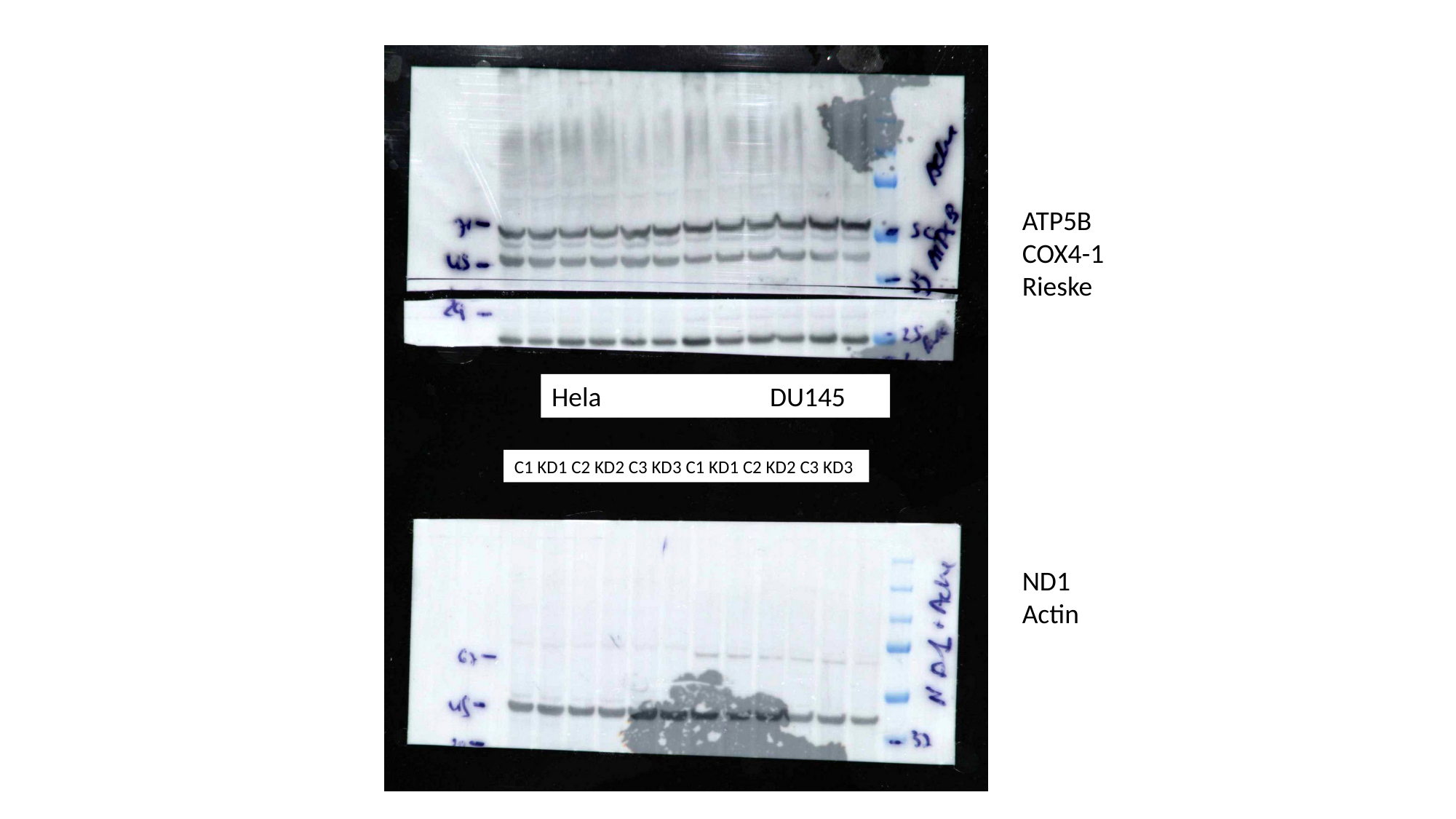

ATP5B
COX4-1
Rieske
ND1
Actin
Hela		DU145
C1 KD1 C2 KD2 C3 KD3 C1 KD1 C2 KD2 C3 KD3

## Slide 20
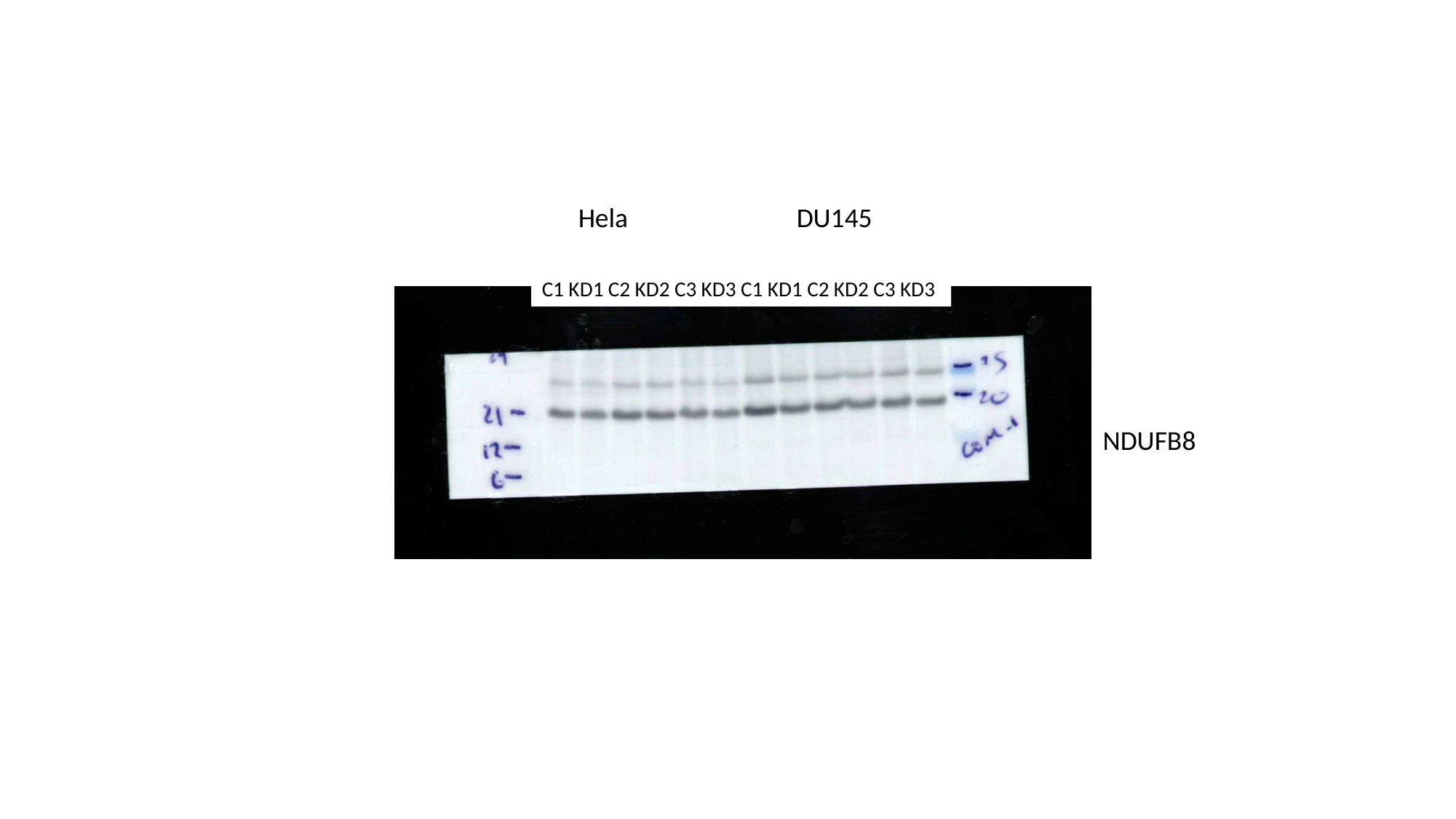

Hela		DU145
C1 KD1 C2 KD2 C3 KD3 C1 KD1 C2 KD2 C3 KD3
NDUFB8
